# Cavernous hemangioma of thymus misdiagnosed as thymoma: a case report

**DOI:** 10.1186/1477-7819-12-323

**Published:** 2014-10-25

**Authors:** Cheng Shen, Yasha Liang, Huan Xu, Lunxu Liu, Guowei Che

**Affiliations:** Department of Thoracic Surgery, West-China Hospital, Sichuan University, Chengdu, 610041 China; Department of General Practice, West-China Hospital, Sichuan University, Chengdu, 610041 China; Department of Pathology, West-China Hospital, Sichuan University, Chengdu, 610041 China

**Keywords:** cavernous hemangioma, surgery, thymus

## Abstract

**Introduction:**

Cavernous hemangioma in the thymus is a rare presentation in mediastinal hemangiomas. The diagnosis is difficult to make promptly because both invasive and noninvasive examinations usually fail to distinguish it from other tumors of the mediastinum. Their clinical presentations depends on their size and their involvement with adjacent mediastinal structures.

**Case presentation:**

We treated a 52-year-old man with thymic cavernous hemangioma that was incidentally detected by chest radiography during a routine health check, and had been misdiagnosed as thymoma before the operation. The tumor was completely resected by thymectomy via video-assisted thoracic surgery. The pathological tissue was diagnosed as a cavernous hemangioma, and no phlebolith was observed in the center.

**Conclusions:**

We reported this case of thymic cavernous hemangioma for its extremely rare occurrence in the thymus. The preoperative diagnosis remains a challenge both clinically and radiologically. It is still difficult to distinguish this tumor from other tumors in the thymus. Furthermore, biopsies might not result in a definitive diagnosis. Finally, surgical resection provides material for histopathologic diagnosis. To facilitate the preoperative diagnosis of such a rare tumor, more cases will need to be reported.

## Background

Cavernous hemangioma is a rare tumor of the mediastinum, accounting for 0.5% or less of all mediastinal tumors [1]. The diagnosis is difficult to make promptly because both invasive and noninvasive examinations usually fail to distinguish it from other tumors of the mediastinum. Their clinical presentations depend on their size and their involvement with adjacent mediastinal structures. We herein report a case of an unusual hemangioma of thymic origin and a review of the literature concerning the clinical and pathological features of this disease, which is often misdiagnosed as thymoma.

## Case presentation

A 52-year-old man was referred to our hospital for assessment of a suspicious mass that was detected on a chest radiography during a routine health check. He denied symptoms, including chest pain, hoarseness, hemoptysis, cough, dry cough, and dyspnea. He was a nonsmoker and had no exposure to any environmental fumes or dust.

**Figure 1:**
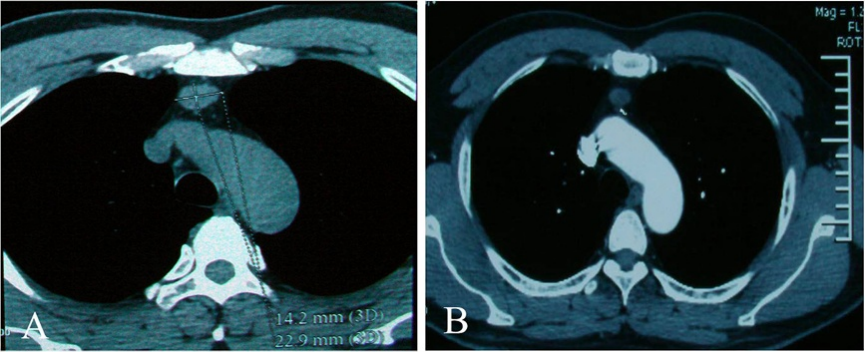
**Computed tomography showed a tissue mass in the anterior mediastinum. (A)** A plain chest scan showed a circumscribed soft tissue mass measuring 2.2 × 1.4 cm in size. **(B)** A contrast-enhanced scan showed a demarcated tissue mass with no calcification.

Physical examination revealed normal breathing sounds in both lung fields. Laboratory findings were within normal limits. In these examinations, hematology test results and biochemistry test results were within regular levels. Plain chest computed tomography (CT) displayed a circumscribed soft tissue mass that was located in the anterior mediastinum, measuring 2.1 × 1.4 cm (Figure 1A). Contrast-enhanced CT showed a demarcated soft tissue mass with no calcification and marginal enhancement (Figure 1B). The lesion was suspected to be a thymoma rather than a vascular neoplasm before surgery.

**Figure 2:**
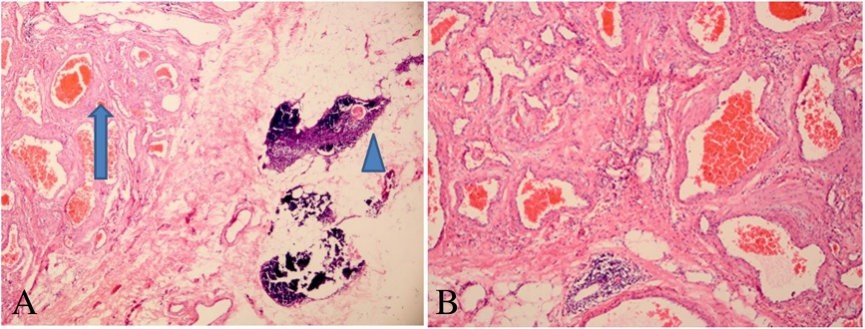
**Histological features. (A)** The channels that are dilated vessels with endothelial cells contained red blood cells (arrow) and a thymic corpuscle (arrowhead) is observed. (H & E 40×). **(B)** Section reveals dilated vessels with endothelial cells. Most of the channels contained red blood cells in the cavity thrombus. (H & E 200×).

As a diagnosis was not established through imaging, surgery was scheduled. We approached the tumor, which was removed by thymectomy using video-assisted thoracic surgery (VATS) rather than a median sternotomy. The thymectomy was performed in the lateral decubitus position, utilizing a three-port, right-sided VATS approach under general anesthesia administered through a double-lumen endotracheal tube. Three entry ports were made at the third and sixth intercostal spaces of the right posterior axillary, midaxillary, and midclavicular lines. The camera was introduced via trocar through the lower port at the sixth intercostal space of the midaxillary line. The operative procedure involved removal of the entire thymus gland including the anterior mediastinal fat, with careful attention to preservation of the phrenic nerve and control of thymic venous tributaries draining into the left brachiocephalic vein. There was no invasion into adjacent structures. After careful attention to hemostasis, a chest tube was placed to drain the pleural cavity, and the right lung was reinflated under direct vision. Macroscopically, examination of the resected specimen showed an encapsulated tumor measuring 2.2 × 1.2 × 0.8 cm in the thymus that could be palpated as a soft nodule. The cut surface seemed to have some fresh blood and the soft tissue was taupe, and no phlebolith was discerned in the center. Histopathologically, it was diagnosed as a cavernous hemangioma consisting essentially of a large number of dilated vessels with a single layer of endothelial cells with no signs of atypia or mitosis. Most of the channels contained red blood cells in the cavity (Figure 2). No tumor tissue was detected at the surgical margin.

The postoperative course was ordinary. The patient was discharged 3 days after the operation with no complication. He has been followed up for 8 months without evidence of recurrence.

## Discussion

Hemangiomas in the mediastinum are uncommon tumors. About 90% of benign blood vascular neoplasms in the mediastinum are cavernous or capillary hemangiomas [2]. Vascular proliferations of the mediastinum include arteriovenous hemangiomas, venous hemangiomas, glomus tumors, hemangiomas, angiofibromas, hemangiopericytomas, and angiolipomas [2]. Cavernous hemangiomas are venous hemangiomas. Although the origin of these tumors in the mediastinum is variable, a small number are of proven thymic origin. Wychulis and colleagues reported a large review of 1,064 cases of mediastinal tumors collected over a 40-year period. Five of these cases were hemangiomas, but none of them was of thymic origin [3]. Yamazaki *et al.* [4] found a total of 61 cases of mediastinal hemangioma reported in Japan over the previous 50 years. Only one of them was of thymic origin. The patient was a 61-year-old man who was also assessed for a suspicious mass identified on a chest radiograph during a routine health check. The mass was a well-demarcated nodule in the left lobe of the thymus. There are three reports of six thymic hemangiomas for which no pathology reports and specific clinical data were available [1, 5, 6].

Yamazaki *et al.* [4] reported that the tumor was asymptomatic in 56% of patients. The lesion might provoke symptoms if it is infectious or if it exerts pressure on neighboring structures after becoming expanded. Patients with cavernous hemangioma present with cough, chest pain, dyspnea, hemoptysis, and hoarseness. It has occasionally been observed as recurrent pleural effusion [7] or recurrent pneumonia caused by an airway obstruction [5]. In our case, the patient presented without any symptoms.

The preoperative diagnostic evaluation for mediastinal tumors consists of an extensive medical history and chest CT. Hemangiomas are habitually round and lobulated with smooth margins. Cavernous hemangiomas may show characteristic ‘puddles’ of enhancement following administration of a contrast agent [7]. According to McAdams *et al.* [8], the center of the tumor shows more enhancement than the margin on CT. In our case, we could not observe this detection. In mediastinal hemangiomas, the occurrence of characteristic ring calcification has been reported in about 10% of cases [9]. However, no calcification was found in our case.

Histopathologically, a cavernous hemangioma is composed of dilated vessels covered by one layer of endothelial cells. Differential diagnosis of the mass includes other tumors in the thymus, such as cystic lymphangioma, angiolipoma, and thymoma. Cystic lymphangioma can occur in any location where normal lymphatic ducts are found. The most frequently reported sites are the head and neck (75%), the axilla (20%), and other organs (5%). Histologically, any dilated lymphatic spaces are lined with attenuated endothelial cells resembling the cells that line normal lymphatics. The lymphatic spaces are usually filled with proteinaceous eosinophilic fluid. The supporting stroma is composed of collagen and may contain lymphocytes and lymphoid aggregates. Angiolipoma is a rare benign tumor consisting of mature adipose tissue and blood vessels. Thymomas are classified using the Masaoka staging system, including stages I, II, III, and IV, and the new World Health Organization schema (A, AB, B1, B2, B3).

As the mass was in the thymus, we considered thymectomy necessary to extirpate the lesion completely and that extirpation by VATS might be a better option to treat this condition than median sternotomy. Surgical resection is associated with excellent long-term prognosis (Table 1).

**Table 1:** **Characteristics of patients**

Patient	Age	Sex	Symptoms	Localization	Size (cm)	Resection	Follow-up (months)	Prognosis
1	35	Male	Pain in neck	Cervical ectopic thymus tissue	3 × 3	Right thoracotomy	24	Alive [2]
2	52	Female	None	Left lobe of thymus	4.2 × 3.2	Right thoracotomy	96	Alive [4]
3	63	Male	Dyspnea	Left lobe of thymus	6 × 6	Left thoracotomy	44	Death [7]
4	59	Female	None	Left lobe of thymus	5 × 4	VATS	26	Alive [9]
5	52	Male	None	Right lobe of thymus	2.1 × 1.4	VATS	12	Alive

VATS, video-assisted thoracic surgery.

## Conclusions

We reported this case of thymic cavernous hemangioma for its extremely rare occurrence in the thymus. Preoperative diagnosis remains a challenge both clinically and radiologically. It is still difficult to distinguish the disease from other tumors in the thymus. Furthermore, biopsies may not result in a definitive diagnosis. Finally, surgical resection provides material for histopathologic diagnosis. To facilitate the preoperative diagnosis of such a rare tumor, more cases will need to be reported.

## Consent

Written informed consent was obtained from the patient for the publication of this case report and accompanying images. A copy of the written consent is available for review by the editor-in-chief of this journal.

## Abbreviations

CT: computed tomography
H & E: hematoxylin and eosin
VATS: video-assisted thoracic surgery.
